# Characteristic Poses in Ballet in the Case of a Retired Classical Ballerina after Bilateral THA: A Case Report and Review of Literature

**DOI:** 10.1155/2021/5560581

**Published:** 2021-06-02

**Authors:** Futoshi Morio, Shigeo Fukunishi, Tomokazu Fukui, Makoto Kanto, Kenta Amai, Shinichi Yoshiya, Toshiya Tachibana

**Affiliations:** ^1^Department of Orthopaedic Surgery, Hyogo College of Medicine, Japan; ^2^Nishinomiya Kaisei Hospital, Japan

## Abstract

**Background:**

Ballet dance involves extreme range of motion (ROM) in the hip joint. This ROM can cause injuries including labral strain, tears, and subluxation episodes. *Case Presentation*. A 69-year-old female classical ballet dance instructor presented bilateral hip pain. The plain radiograph showed end-stage osteoarthritic change in the bilateral hip. She could neither dance nor perform daily activities. Bilateral hybrid total hip arthroplasty (THA) was performed. After surgery, she was able to demonstrate the split position on the floor as an active classical ballerina. The plain radiograph was taken in the split position, and the radiograph did not show any characteristics of impingement or subluxation of the femoral head.

**Conclusion:**

She was able to continue working as a classical ballet instructor after bilateral THA. Additionally, any characteristics of impingement or subluxation of the femoral head were not revealed in the postoperative radiograph in the split position.

## 1. Introduction

Ballerinas are often described as both artists and athletes. In recent years, the number of people who learn ballet has increased worldwide. Ballerinas' lower extremities leave them at risk of musculoskeletal injuries due to the unique demands in ballet. Previous studies have reported injury incidence rates of 67% to 95% among professional ballet dancers and 17% to 24% in modern dancers [1–6]. Ballet dance involves extreme hip abduction, flexion, and extension, as well as external rotation (i.e., turnout). This extreme range of motion can cause injuries, including labral strain, tears, and subluxation episodes [7, 8]. Hip injuries have been reported in multiple studies and comprises between 7% and 50% of injuries in dancers [9]. In a recent systematic review, hip/groin injuries in professional dancers were recorded, at a rate of 27.7% [10]. There are few reports on performing total hip arthroplasty (THA) for ballerinas, as many orthopedic surgeons tend to choose arthroscopic or osteotomy surgery. Because of the risk of dislocation after THA, many orthopedic surgeons may hesitate to do THA. In this report, we present a rare case of a ballet dance instructor who sustained bilateral osteoarthritis of the hip and underwent bilateral THA. She was able to successfully go back to her previous dance activities after surgery. The purpose of this article is to describe the management of ballerina who sustained end-stage hip osteoarthritis. If surgeons understand the characteristic movement of a ballerina, we are considering that THA for ballerina is not a contraindication.

## 2. Case Presentation

This study was approved by the Institutional Review Board of Hyogo College of Medicine, and informed consent was obtained from the patient in the study.

**Figure 1:**
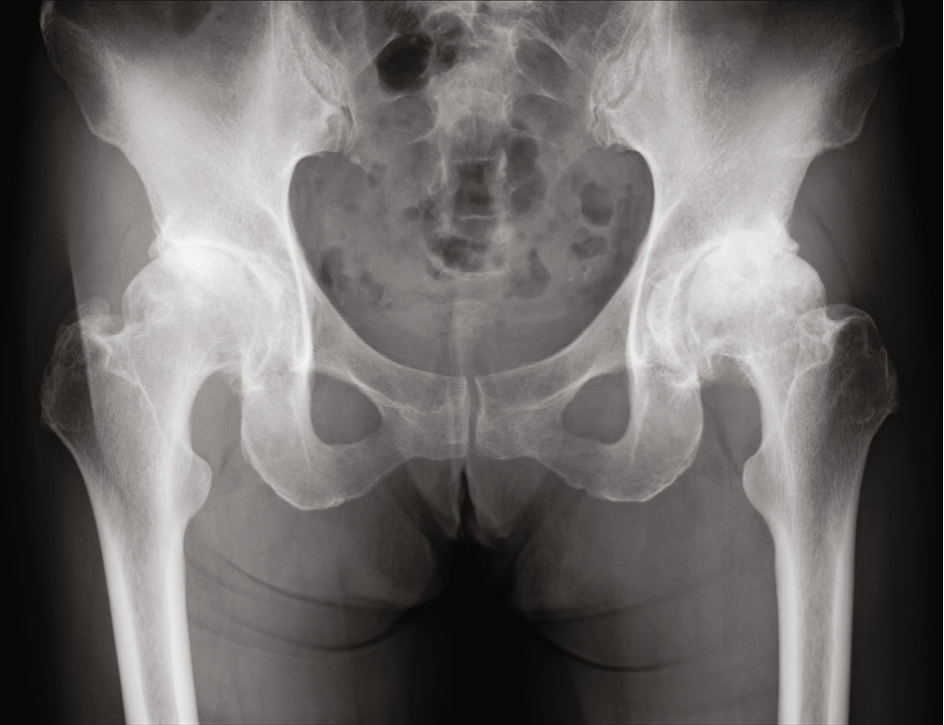
The plain radiograph of initial visit to our hospital at 7 years before surgery. Osteoarthritic changes in both hips without acetabular dysplasia were identified.

**Figure 2:**
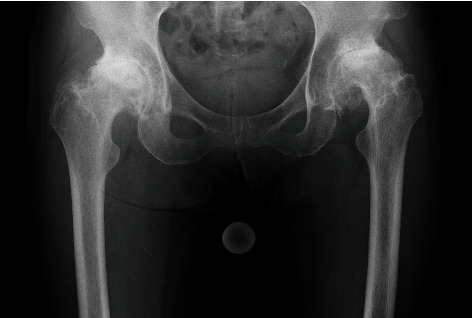
The plain radiograph before surgery. End-stage osteoarthritis was revealed in both hips.

**Figure 3:**
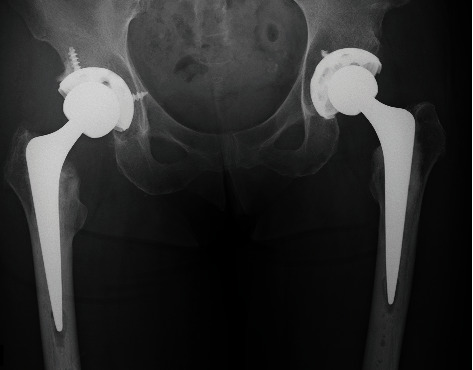
Postoperative plain radiograph. Hybrid THA was performed on both hips.

**Figure 4:**
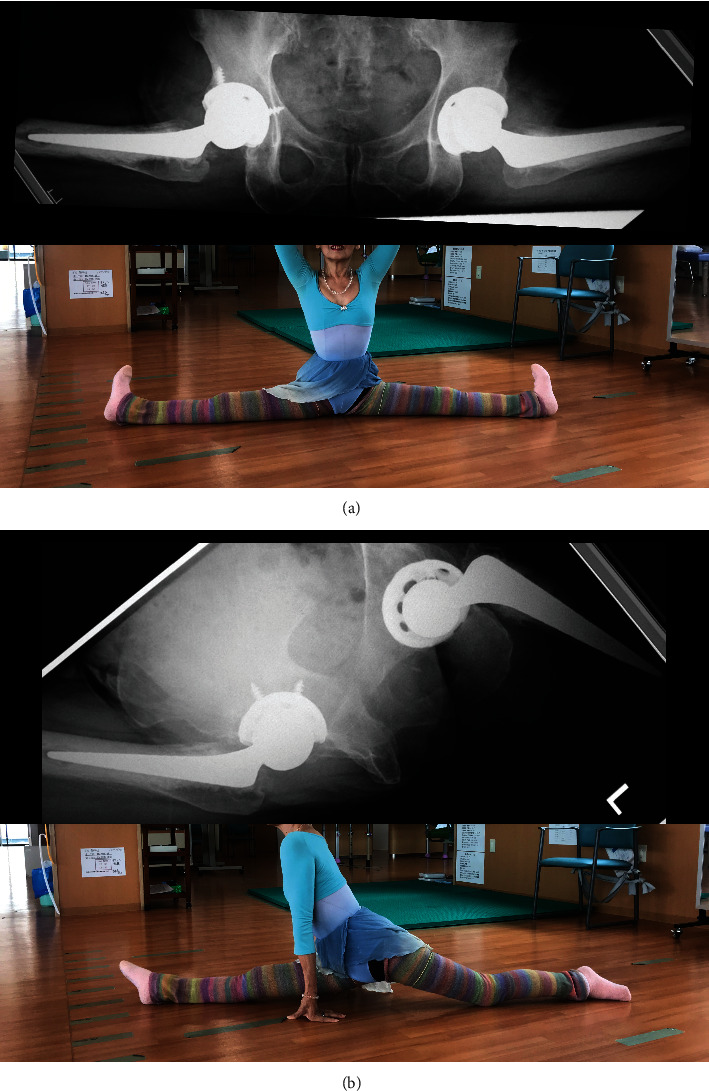
Postoperative radiograph with two types of characteristic postures in ballet. The patient was demonstrating the split position. These views were obtained with cassettes by getting as close as possible to the patient's back during the actual postures. The radiograph did not show any characteristics of bony impingement, implant-implant impingement, or subluxation of the femoral head: (a) split position; (b) left split position.

A 69-year-old female ballet dance instructor presented discomfort and pain in her bilateral hip. She had danced ballet since childhood, had appeared on many stages, and played an active role as a professional ballerina. She noted the symptoms approximately 3 years before her initial visit to our institute without a history of hip trauma. Additionally, she had no other family history of acetabular dysplasia. Her initial physical examination revealed bilateral hip pain (L > R) with limitation in the range of motion (ROM). Hip pain was exacerbated by ballet movements, but it was possible to dance on the stage. The plain radiograph showed osteoarthritic change in both hips, and borderline acetabular dysplasia was suspected as a sharp angle (R°/L°) of 39°/41°, a center edge angle (R°/L°) of 25°/23°, and acetabular head index (R %/L %) of 82%/80% (Figure 1). Seven years after the initial visit, hip pain and limitation in ROM (R°/L°) progressed as follows: flection 100°/90°, external rotation 45°/30°, internal rotation 0°/0°, and abduction 20°/15°. The plain radiograph revealed end-stage osteoarthritis (OA) (Figure 2). At that time, she could neither dance nor perform daily activities. She had retired from being a ballerina and became an instructor. THA was performed on the left hip first, and subsequently, THA was performed on the right hip 9 months after the initial surgery. Both THA were performed with the modified Hardinge approach in the lateral position. A computed tomography- (CT-) based navigation system (CT-based hip navigation version 1.1, Stryker Navigation, Freiburg, Germany) was assisted for cup positioning. A cementless cup (Trident Acetabular Shell, Stryker Orthopedics, NJ, USA), a cemented stem (Exeter V40 Femoral Stem, Stryker Orthopedics, NJ, USA), a ceramic head (BIOLOX delta V40 Ceramic Head, Stryker Orthopedics, NJ, USA), and a nonelevated ultrahigh molecular weight polyethylene liner (Trident X3 insert, Stryker Orthopedics, NJ, USA) were implanted. For preoperative planning of cup placement in our surgical concept, radiographic cup inclination was fixed at 40 degrees while radiographic cup anteversion was aimed at approximately 20 degrees. However, sufficient cup coverage in the original acetabulum based on individual anatomy is given priority over the general target anteversion to ensure adequate cup coverage. Following the theory, preoperative target cup alignment is proceeded to prioritize avoiding anterior or posterior protrusion from acetabular edge over the target radiographic anteversion angle [11]. So, in the present case, preoperative planning was inclination 40° and anteversion (R°/L°) 22°/28°. During surgery, to avoid the implant-implant and implant-bone impingement and subsequent dislocation, we adjust stem anteversion using a cemented stem depending on the cases. Impingement was confirmed through the impingement test with flexion 120°, flexion 90° with internal rotation 60°, and extension 0° with maximum external rotation by the trial stem as our routine procedure. The implant-implant impingement and the bony impingement were not revealed. For the leg length adjustment, we gave priority to the joint stability in order to avoid impingement rather than leg lengthening. In the present case, we planned bilateral THA subsequently, and equalization of leg length could be achieved at the time of the second surgery (Figure 3). After implantation, the anterior capsule was repaired. The postoperative rehabilitation program was not special for the ballerina, and she underwent free mobilization and full-weight-bearing exercise one day after surgery. At the time of THA, we did not expect the patient to be able to give demonstrations of ballet dance during the coating period. However, three years after surgery, she was able to continue working as an instructor. The ROM had improved as follows: flection 110°/115°, external rotation 45°/45°, internal rotation 30°/30°, and abduction 45°/45°. Amazingly, the patient was able to demonstrate the split position on the floor as an active classical ballerina (video (available here)). The plain radiograph was taken in the split position (Figure 4(a)) and left split position (Figure 4(b)), and the radiograph did not show any characteristics of impingement or tendency of dislocation.

## 3. Discussion

Ballet is a healthy activity which helps people to improve their quality of life. On the other hand, ballet is a “high-risk” activity with high incidence of musculoskeletal impairments. Duthon et al. and Jacobs et al. described that extreme range of motion of the hip can cause injuries, including labral strain, tears, and cartilage damage in the superior and posterosuperior areas of the acetabulum [7, 8]. The years of dancing can contribute to a ballerina developing pain secondary to OA in the hip due to repetitive extreme movements [12]. It has been reported that the practice of some dance movements could expose the dancer's hip to loss of joint congruence and recurrent impingements, which could lead to early OA. In the present case, it could be possible that borderline acetabular dysplasia participates in the progressed osteoarthritis; however, the repetitive extreme movements and the recurrent impingements in the characteristic motion of a ballerina could cause progressed osteoarthritis. Additionally, Buyls et al. [13] described that left hip pain was involved in all dancers with unilateral OA, which may reflect the tendency of ballerinas to use the left leg as the standing leg, and suggest that strenuous physical activity may lead to osteoarthritis. Just as in the present case, OA of the left hip was more progressed than that of the right hip. In surgical treatment of a hip disorder for the ballerina, when hip pain develops in a ballerina and conservative treatment fails, the most common decision to be made by surgeons is whether the patient is a candidate for arthroscopic treatment of the hip or requires an open procedure, such as periacetabular osteotomy [14, 15]. Although there are many hip disorders in ballerinas epidemiologically, there are only two case reports on performing THA on ballerinas. This may be because many orthopedic surgeons are concerned about the risk of dislocation caused by implant-implant impingement and bony impingement due to the wide range of motion. Recently, Komiyama et al. [16] analyzed the dynamic hip kinematics of patients with recreational classical ballet who underwent THA and reported that no implant impingement was revealed during two typical postures. Regarding the characteristic poses and hyperflexibility of the hips in ballerinas, “turnout” is emphasized; this is the hip in external rotation with the knees in extension in ballet. The technique of classical ballet is based on “turnout” or outward rotation of the legs. Therefore, the movements of classical ballet must be executed in a “turnout” position. The ideal “turnout” demonstrates 180° of external rotation starting at the hips and results in the feet being easily placed in a 180° position on the floor. Therefore, ballerinas have increased range of motion of the hip in external rotation and decreased internal hip rotation compared to nondancers [17]. Normally, when measuring the abduction angle, the leg to be measured was maintained in a neutral rotation with the foot perpendicular to the floor. It was then abducted passively in the frontal plane to the extreme of range [18]. With this measurement method, the abduction angle showed to be lower than 45°. However, in ballerinas, hip abduction is combined with “turnout” and hip flexion is a movement used by ballet dancers during many movements at a standing position. Valenti et al. [19] stated that the more the dancer externally rotates the hip, the greater the hip abduction will be. In the present case, postoperative hip ROM was measured and showed flexion 110°/115°, external rotation 45°/45°, internal rotation 30°/30°, and abduction 45°/45° on the bed for medical examination. These values show a wider ROM as compared to normal patients, and we could not recognize the extreme wide range of motion of ballerinas; however, the patient was able to demonstrate her range of split position.Surgeons and physical therapists should understand characteristic movements of ballerinas during surgery and physical therapy and keep in mind that ballerinas could achieve the nearly 90° abduction by combining maximum external rotation. As the matter of course, both soft tissue preservation and precise implant positioning are important to avoid impingement and dislocation for THA. On the other hand, we consider that a strict impingement test during surgery according to the characteristic posture of a ballerina which is hyperextension and hyperabduction with external rotation could be the important key management rather than combined anteversion as generally recommended. The postoperative radiograph with the patient in the split position showed the relationship of the acetabulum, femur, and implant. In this radiograph, the femur was externally rotated and the major trochanter takes the bottom direction. It could avoid the impingement between the greater trochanter and the posterior acetabular rim. Additionally, implant-implant impingement did not occur in this characteristic posture. Generally, orthopedic surgeons hesitate to do THA considering the risk of dislocation due to the wide range of motion in a ballerina. However, we could understand that characteristic movements of ballerinas with nearly 90° abduction by combining maximum external rotation is not a risk for impingement unexpectedly. On the other hand, Figure 4(b) shows the right hip in flexion with the knees in extension anteriorly and left hip hyperextended with the knee extended posteriorly. The radiograph did not show implant-implant impingement or subluxation of the femoral head. However, if the left hip will be hyperextended perfectly, it is possible that the liner-to-neck contact might develop. Following the results, implant-implant impingement and subluxation of the femoral head should be checked by conducting an impingement test with hyperextension of the hip, knee, and ankle in neutral positions during left hip THA for the ballerina.

There were some limitations in this study. First, the patient in the present study was a 69-year-old retired ballerina; therefore, the results may differ and the indication for THA may not be necessary for an active and younger ballerina. Age, sex, performance level, and pelvic alignment could be other factors for the indication of THA. Therefore, we could not recommend THA for all ballet dancers. Second, since only static analysis was performed in the present study, it is necessary to perform dynamic analysis in the future. Third, the postoperative follow-up period was quite short in this case report. The patient's wide range of motion and activity after THA might pose a risk of developing dislocation or polyethylene wear in the future. Therefore, future observation of progress is necessary.

## 4. Conclusion

The patient was able to continue working as a classical ballet instructor after bilateral THA. Additionally, any characteristics of impingement or subluxation of the femoral head were not revealed in the postoperative radiograph in the split position.

## Abbreviations

ROM: Range of motion
THA: Total hip arthroplasty
CT: Computed tomography.

## Supplementary Materials

Supplementary Materials Characteristic poses in ballet of this patient after bilateral THA (video).
