# Supplementary material for: Characteristic Poses in Ballet in the Case of a Retired Classical Ballerina after Bilateral THA: A Case Report and Review of Literature
Source: Case Rep Orthop. 2021 Jun 2;2021:5560581. doi: 10.1155/2021/5560581 (PMC8189802; doi:10.1155/2021/5560581)
Supplement: Supplementary Materials — Characteristic poses in ballet of this patient after bilateral THA (video). [file 5560581.f1.pdf]

Characteristic poses in Ballet
in the case of a retired classical ballerina after Bilateral THA
-A cases report and review of literature-

Supplemental Files

Characteristic poses in ballet of this patient after bilateral THA. (Video)

The patient was able to demonstrate the split position on the floor as an active classical ballerina

https://drive.google.com/file/d/1q4lZ_W_Z6rHvbYZwHk44fp1_FZ5tcAWF/view?usp=sharing

Hyogo College of Medicine
1-1 Mukogawa-cho
Nishinomiya City, Hyogo, Japan 663-8501
Tel: 81-798-45-6452 Fax: 81-798-45-6453
e-mail: morio1223.com@gmail.com
